# Association of OGTT-curve Shape With Anthropometric, Metabolic, and Inflammatory Parameters in Healthy Adults

**DOI:** 10.1210/jendso/bvaf060

**Published:** 2025-04-07

**Authors:** Lisa Sophie Fischer, Eva Kiesswetter, Beate Brandl, Thomas Skurk, Hans Hauner, Dorothee Volkert, Robert Kob

**Affiliations:** Institute for Biomedicine of Ageing, Friedrich-Alexander-Universität Erlangen-Nürnberg, 90431 Nuremberg, Germany; Institute for Biomedicine of Ageing, Friedrich-Alexander-Universität Erlangen-Nürnberg, 90431 Nuremberg, Germany; Institute for Evidence in Medicine, Medical Center and Faculty of Medicine, University of Freiburg, 79110 Freiburg, Germany; ZIEL—Institute for Food & Health, Technical University of Munich, 85354 Freising, Germany; ZIEL—Institute for Food & Health, Technical University of Munich, 85354 Freising, Germany; Else Kroener Fresenius Center of Nutritional Medicine, Technical University of Munich, 85354 Freising, Germany; Else Kroener Fresenius Center of Nutritional Medicine, Technical University of Munich, 85354 Freising, Germany; Institute of Nutritional Medicine, School of Medicine and Health, Technical University of Munich, 80333 Munich, Germany; Institute for Biomedicine of Ageing, Friedrich-Alexander-Universität Erlangen-Nürnberg, 90431 Nuremberg, Germany; Institute for Biomedicine of Ageing, Friedrich-Alexander-Universität Erlangen-Nürnberg, 90431 Nuremberg, Germany

**Keywords:** OGTT, GLP-1

## Abstract

**Introduction:**

The shape of the oral glucose tolerance test (OGTT) curve is an early predictor of metabolic disturbances. In this study, we analyzed which parameters are associated with different OGTT-curve shapes (CS) in healthy middle-aged and older adults.

**Methods:**

In the cross-sectional Enable Study, 354 participants were comprehensively phenotyped. Based on a 2-hour OGTT, CS was classified according to the presence (polyphasic) or absence (monophasic, mp) of a rise in plasma glucose of more than 4.5 mg/dL after the first decline of the plasma glucose level. Associations between CS and age, sex, anthropometric, metabolic, and inflammatory parameters were analyzed by binomial logistic regression.

**Results:**

Curve shape was mp in 77.4% of the participants without age group difference, but a higher frequency was observed in men (89.3%) compared to women (65.5%, *P* < .001). The odds of mp CS increased with higher fasting GLP-1 (odds ratio [OR], 1.066; 95% CI, 1.006-1.133; *P* < .05) and 1-hour plasma glucose (OR, 1.054; 95% CI, 1.037-1.072; *P* < .001) and lower 2-hour plasma glucose (OR, 0.975; 95% CI, 0.959-0.992; *P* < .01).

**Conclusion:**

In healthy adults, mp CS was widespread and associated with more unfavorable metabolic parameters. A higher fasting GLP-1 level was associated with an mp CS.

The oral glucose tolerance test (OGTT) is an established tool for assessing glucose metabolism in clinical settings [1]. Most commonly used parameters of the OGTT are fasting (FPG) and 2-hour plasma glucose levels (2h-PG) [1, 2]. However, several studies concluded that these criteria often fail to identify the prediabetic stages [2, 3]. Recent studies have reported that the shape of the OGTT curve is more sensitive to early states of ß-cell dysfunction and insulin resistance than FPG and 2h-PG [4-6].

OGTT curve shapes (CS) can be categorized as monophasic (mp), biphasic, and sometimes also polyphasic (pp) [4]. The characteristic of a monophasic shape is the absence of a second rise of the plasma glucose level of more than 4.5 mg/dL after the first decline, whereas the presence of such an increase classifies the curve as biphasic [7, 8]. CS with 2 and 3 peaks are summarized as pp [9]. An mp CS reflects an increased risk of a reduced glucose tolerance as well as a lower insulin sensitivity and worse ß-cell function in adolescents and adults [4, 7, 9-11]. Most recent studies demonstrated also an incessant increase CS type, marked through the absence of a decrease of the plasma-glucose curve during the observation period, indicating an even worse ß-cell function and insulin sensitivity up to manifest diabetic conditions [12, 13].

To assess the future metabolic risk in terms of an impaired glucose metabolism, only very limited data are available, showing a higher prevalence and higher degree of metabolic dysfunction associated with mp curves [14]. Additionally, mp CS has been linked to higher levels of inflammatory parameters like C-reactive protein (CRP) and IL-6 [6, 15]. To date, knowledge about mp CS comes mainly from adolescents [10, 11] and middle-aged adults [4, 8, 10], whereas only 1 study examined persons aged older than 65 years [16].

A better knowledge of the relationship between OGGT CS and metabolic and inflammatory parameters could help to assess the impact on cardiovascular risk, which is severely increased in metabolic syndrome (metS) [17]. In this study, we examined the prevalence of mp OGTT CS in healthy middle-aged and older adults. In addition, participants were comprehensively phenotyped to identify anthropometric, metabolic, and inflammatory parameters associated with mp CS.

## Methods

### Study Design and Participants

In this secondary data analysis from the Enable Study, a cross-sectional study that was performed in healthy, community-dwelling persons in 2 study centers in Southern Germany between February 2016 and February 2018 [18], we examined OGTT data along with anthropometric, metabolic, and inflammatory parameters. Persons aged 40 to 65 years were recruited in Freising with the aim to enroll ∼50% of participants with increased waist circumference (>88 cm women, >102 cm men). Persons aged 75 to 85 years were recruited in Freising and Nuremberg. The aim of the recruitment strategy in the main Enable Study was to represent a middle-aged, working group and an older group with beginning functional restrictions for substudies dedicated for these stages of life. For both age groups, an equal gender proportion was intended.

Exclusion criteria for both age groups were immobility, food allergies and intolerances, pregnancy, unintentional weight loss of more than 5% of bodyweight in the past 3 months, acute diseases, and diagnosed chronic diseases (eg, diabetes mellitus, autoimmune diseases, uncontrolled hypertension >160/95 mm Hg). Infections of the upper airways, lung diseases, diagnosed neurologic or psychologic disorders in the past 3 years, renal failure requiring dialysis, stomach ulcers, liver diseases, endocrinological diseases as well as coronary artery disease, a history of myocardial infarction or stroke, cancer, and active smoking also entailed exclusion. For the older age group, an additional exclusion criterion was need of care according to SGB §XI (German code of social law) [15].

### Anthropometric Parameters

Anthropometric data were measured in the study centers in the morning after overnight fasting.

Body height was measured without shoes in a standing position using a stadiometer (Seca 213, Seca GmbH&Co. KG, Hamburg, Germany). Body weight (BW) and body composition were measured by a bioelectrical impedance analysis device (Seca medical Body Composition Analyzer, mBCA 515, Seca GmbH&Co. KG). BW was measured in light clothes without shoes and corrected for clothes by subtracting 1 kg. Body mass index (BMI) was calculated by dividing BW by the squared body height (kg/m^2^). From bioelectrical impedance analysis, fat mass (kg) and fat-free mass (kg) were collected and the percentage of body fat and fat-free mass calculated.

Waist circumference (WC) was measured according to the World Health Organization guidelines; WC ≥ 80 cm for women and ≥94 cm for men were defined as increased [19].

### Metabolic and Inflammatory Parameters

Venous blood samples were collected after overnight fasting into vacutainers added with lithium heparin, sodium fluoride, and EDTA with gel (S-Monovette, Sarstedt AG&CO., Nümbrecht, Germany). For serum collection, the vacutainer without anticoagulants was left for 30 minutes before centrifugation at 2500*g* for 20 minutes at 20 °C. The EDTA-containing vacutainers were centrifuged immediately at the same conditions for plasma separation. Plasma and serum were aliquoted and stored at −80 °C for further analysis.

FPG, insulin, triglycerides (TG), total cholesterol, high-density lipoprotein (HDL) cholesterol, low-density lipoprotein cholesterol, alanine-amino-transferase (ALT, also known as glutamate-pyruvate-transaminase [GPT]), ferritin, high-sensitivity CRP and TSH were analyzed by a certified laboratory (Synlab Laboratory Center Munich or Nuremberg, Germany) using routine methods.

Total GLP-1 was measured in EDTA-plasma with the Total ELISA 96-Well Plate Assay (GLP1T-36HK, RRID:AB_2757816, EMD Millipore Corporation, St. Louis, Missouri, USA). Leptin and IL-6 were quantified using Human Magnetic Luminex Assays (LOBM1786, RRID:AB_3099472, R&D Systems Europe Ltd., Abingdon, UK) according to the supplier’s instructions.

### Participants´ Health Status

Blood pressure (BP) was measured on both upper arms once plus another 2 times on the arm with higher BP, using Omron M8 comfort/M300 (omron Medizintechnik, Mannheim, Germany) or Maxi Stabil 3 (WelchAllyn GmbH&Co. KG, Hechingen, Germany). The mean value of the 3 measurements was calculated and used for further analysis.

Metabolic syndrome was defined according to the International Diabetes Federation definition as increased WC (>80 cm women, >94 cm men) plus at least 2 of the following 4 criteria: TG ≥ 150 mg/dL or premedication with lipid-lowering agents, HDL cholesterol <50 (women), <40 mg/dL (men), FPG ≥ 100 mg/dL or diagnosed type 2 diabetes mellitus, which was in fact an exclusion criterion for our study, systolic BP ≥ 130 mm Hg or diastolic BP ≥ 85 mm Hg or antihypertensive medication [19].

### OGTT Curve Shapes

Following fasting blood sampling, participants consumed a drink of 75 g glucose (Carl Roth GmbH&Co. KG, Karlsruhe, Germany) in 300 mL of water. Capillary blood was drawn just before and at 30, 60, 90, and 120 minutes after glucose intake. Plasma glucose concentrations (mg/dL) were quantified immediately by using the HemoCue Glucose 201+ System (HemoCue, plasma-calibrated, Ängelholm, Sweden). OGTT curves were created by connecting the single blood glucose concentrations at the 5 time points. The area under the glucose curve was calculated for 0 to 120 minutes (AUCglucose 120 minutes) using the trapezoid method with zero glucose as baseline [20].

OGTT CS was categorized as mp or pp depending on the absence or presence of an additional rise of the glucose level of more than 4.5 mg/dL after the first decline. Additionally, it was analyzed whether participants showed a continuously rising curve over the whole time.

Insulin sensitivity was calculated by using the homeostasis model assessment for insulin resistance (HOMA-IR) [21].

### Data Analysis and Statistics

*t*-tests were used to compare continuous variables by sex within each age group and by age groups of same sex. The chi-squared test was used to compare participants with mp and pp CS by sex, age group, single metS criteria (WC, TG, HDL cholesterol, FPG, BP), and metS in total.

Due to the sufficient sample size and the absence of extreme outliers (defined by a deviation of more than 3 times greater than the interquartile range) unequal variance *t*-test was used to compare anthropometric, metabolic, and inflammatory parameters of participants with mp and pp CS.

Factors that were significantly different between mp and pp CS in the univariate analysis were further evaluated in a multivariate binominal logistic regression model adjusted for age and study center. Due to excessive collinearity, HOMA-IR instead of basal insulin and FPG, and 1-h and 2-h plasma glucose instead of AUCglucose were used in the final model. As sensitivity analyses, the same model was calculated limited (1) to participants without impaired FPG and additionally (2) for participants without metS.

Data were analyzed using IBM SPSS Statistics 29 (IBM Corp., Armonk, USA). *P* values < .05 were regarded as statistically significant.

## Results

Data of 354 participants (63.8 ± 13.9 years, 50.0% women)—198 middle-aged (52.6 ± 6.9 years, 51.0% women) and 156 older adults (78.2 ± 2.8 years, 48.7% women)—were examined.

Anthropometric, metabolic, and inflammatory characteristics are shown in Table 1 by sex and age group. Triglyceride levels were highest in middle-aged men. Total cholesterol and HDL cholesterol levels were higher in women than in men. Ferritin and GLP-1 levels were lower, and leptin higher in women than in men of both age groups.

**Table 1.** Participant characteristics

	Women (n = 177)		Men (n = 177)	
	40-65 y (n = 101)	75-85 y (n = 76)	40-65 y (n = 97)	75-85 y (n = 80)
Age (y)	51.0 [46.5-56.5]	77.0 [76.0-80.8]	54.0 [48.0-59.5]	77.5 [76.0-80.0]
Anthropometric parameters				
Body weight (kg)	73.3 [61.2-83.9]	62.2 [59.8-74.2]	90.1 [80.7-101.1]	80.9 [72.0-88.7]
Height (cm)	165.7 ± 6.2	160.2 ± 6.6	179.8 ± 5.3	173.0 ± 6.8
BMI (kg/m^2^)	26.6 [21.9-30.9]	25.4 [23.0-28.7]	27.9 [25.3-31.6]	27.5 [24.5-29.3]
Waist circumference (cm)	87.6 ± 13.9	89.5 ± 13.0	100.5 ± 11.5	102.2 ± 11.2
Fat mass (kg)	28.6 [19.3-36.6]	29.0 [22.4-34.1]	25.2 [19.8-32.9]	25.6 [20.4-30.4]
Fat-free mass (% body weight)	61.2 [57.1-67.8]	57.0 [52.6-61.6]	71.9 [68.1-75.8]	68.6 [65.0-72.6]
Metabolic parameters				
Fasting plasma glucose (mg/dL)	92.0 [87.0-97.5]	93.0 [85.0-100.0]	95.0 [87.0-100.0]	98.0 [88.3-103.8]
1-h plasma glucose (mg/dL)	132.0 [109.0-159.5]	141.5 [117.0-169.8]	165.0 [137.0-188.0]	161.6 [128.8-190.8]
2-h plasma glucose (mg/dL)	102.0 [87.0-125.5]	106.0 [94.0-138.0]	102.0 [85.0-127.0]	102.5 [82.3-138.0]
AUC glucose (mg/h/dL)	243.8 [211.3-274.8]	250.8 [215.0-293.3]	268.0 [235.8-315.8]	282.5 [238.0-324.8]
Fasting insulin (µU/mL)	3.20 [2.00-6.55]	3.60 [2.00-5.90]	5.40 [2.15-10.15]	3.50 [2.00-6.48]
HOMA-IR (mg/dL)	0.73 [0.46-1.66]	0.81 [0.49-1.48]	1.09 [0.54-2.31]	0.85 [0.49-1.55]
GLP-1 (pM)	11.9* [9.3-16.2]	13.0* [9.9-16.5]	15.9* [11.7-22.1]	17.4* [13.0-24.2]
Triglycerides (mg/dL)	97.0* [69.5-127.5]	103.0 [75.0-131.8]	125.0*^,^** [83.5-181.0]	97.0** [73.5-133.75]
Total cholesterol (mg/dL)	220.0 [195.0-253.0]	234.5* [206.3-264.8]	214.0 [195.5-241.5]	208.5* [181.8-237.0]
HDL cholesterol (mg/dL)	62.0*^,^** [55.5-78.0]	70.0*^,^** [59.5-80.8]	49.0*^,^** [43.5-57.5]	56.5*^,^** [48.3-66.0]
LDL cholesterol (mg/dL)	139.1 ± 42.7	145.3 ± 38.1	141.4 ± 30.9	131.2 ± 37.9
Leptin (ng/mL)	14.21* [5.22-27.94]	13.71* [7.17-26.54]	8.00* [4.04-12.83]	5.89* [3.27-13.44]
GPT/ALT (U/I)	21.0 [16.0-26.0]	20.0 [16.0-23.0]	29.0 [23.0-37.0]	22.0 [18.0-27.0]
TSH (µU/mL)	1.24 [0.83-1.64]	1.12 [0.71-1.44]	1.46 [0.88-1.87]	1.13 [0.77-1.56]
Inflammatory parameters				
Ferritin (ng/mL)	57.0*^,^** [33.5-111.0]	112.0*^,^** [64.5-183.0]	189.0*^,^** [113.0-268.0]	156.0*^,^** [94.0-251.5]
CRP high sensitive (mg/dL)	0.12 [0.06-0.33]	0.17 [0.09-0.37]	0.13 [0.07-0.28]	0.14 [0.08-0.26]
IL-6 (pg/mL)	2.22 [1.13-3.00]	2.90 [1.31-3.60]	2.36 [1.23-3.16]	2.90 [1.98-3.67]

Median [interquartile range].

Abbreviations: AUCglucose, area under the curve of plasma glucose; CRP, C-reactive peptide; GLP-1, glucagon like peptide-1; GPT/ALT, alanine aminotransferase; HDL, high-density lipoprotein; HOMA-IR, homeostasis model assessment index for insulin resistance; LDL, low-density lipoprotein.

^*^Statistically significant difference between sexes of the same age (*P* < .05).

^**^Statistically significant difference between the age groups of same sex (*P* < .05).

In total, 77.4% of the participants, 74.7% of middle-aged and 80.8% of older participants, were categorized as mp (*P* > .05), significantly more men (89.3%) than women (65.5%, *P* < .001). With the exception of HDL cholesterol (*P* = .247), all metS criteria and, consequently, also metS were significantly more common in participants with mp than with pp CS (Table 2).

**Table 2.** Prevalence of single and combined criteria of metabolic syndrome in participants with mono- and polyphasic curve shape

	Monophasic (n = 274)	Polyphasic (n = 80)
Increased waist circumference (w ≥ 80 cm; m ≥ 94 cm) (n = 261)	211 (77.0%)	50 (62.5%)*
Impaired fasting glucose (≥100 mg/dL) (n = 95)	86 (31.4%)	9 (11.3%)**
Increased triglycerides (>150 mg/dL) and/or treated before (n = 99)	89 (32.5%)	10 (12.5%)**
Decreased high-density lipoprotein cholesterol (women ≤50, men ≤40 mg/dL) (n = 34)	29 (10.6%)	5 (6.3%)
Hypertension: diagnosed and/or treated or 1-time measured at study center (n = 256)	208 (75.9%)	48 (60.0%)*
Metabolic syndrome (n = 136)	118 (43.1%)	18 (22.5%)**

Pearson-Chi-Quadrat-Test.

Statistically significant difference between groups: *P < .01; **P < .001.

**Figure 1.**
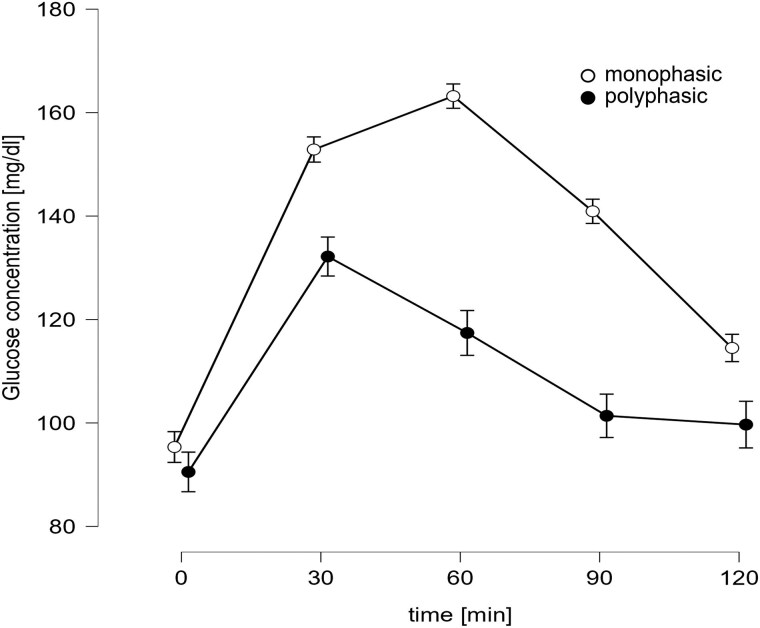
Monophasic and polyphasic OGTT curve shapes. Mean ± standard error of mean of blood glucose concentrations 0 to 120 minutes after ingestion of 75 g glucose in 300 mL water for participants showing either a monophasic (continuous progression of blood glucose level after first rise; n = 274) or polyphasic (additional rise of the blood glucose level of more than 4.5 mg/dL after the first decline; n = 80) curve shape.

Glucose concentrations of participants with mp and pp CS are illustrated in Fig. 1.

While age and percentage of fat free mass were comparable between the mp and pp groups, BMI and waist circumference were significantly higher in the mp group (both *P* < .001, Table 3). All metabolic biomarkers except leptin, total and low-density lipoprotein cholesterol were significantly more unfavorable in persons with an mp CS (all *P* < .001). While ferritin was significantly higher in the mp group (*P* < .001), CRP and IL-6 were similar in the 2 groups.

**Table 3.** Age, anthropometric, metabolic, and inflammatory parameters in healthy middle-aged and older adults with monophasic or polyphasic OGTT curve shape

	Monophasic (n = 274)	Polyphasic (n = 80)
Age (y)	64.4 ± 13.9	62.0 ± 13.6
Anthropometric parameters		
BMI (kg/m^2^)	27.6 ± 4.27	25.0 ± 3.80*
Waist circumference (cm)	97.2 ± 13.7	86.8 ± 11.8*
Fat-free mass (%)	65.9 ± 8.32	65.3 ± 9.16
Metabolic parameters		
Fasting plasma glucose (mg/dL)	95.3 ± 11.3	90.5 ± 8.48*
1-hour plasma glucose (mg/dL)	163.2 ± 40.7	117.4 ± 29.6*
2-hour plasma glucose (mg/dL)	114.5 ± 37.2	99.7 ± 28.7*
AUCglucose 120 minutes (mg/h/dL)	281.0 ± 58.9	223.0 ± 37.7*
Fasting insulin (µU/mL)	6.03 ± 5.24	3.99 ± 3.42*
HOMA-IR (mg/dL)	1.45 ± 1.35	0.90 ± 0.79*
GLP-1 (pM)	16.9 ± 7.65	12.6 ± 4.95*
Triglycerides (mg/dL)	119.8 ± 59.5	97.1 ± 38.9*
Total cholesterol (mg/dL)	222.1 ± 41.4	223.3 ± 39.6
HDL cholesterol (mg/dL)	60.2 ± 16.7	67.0 ± 16.3*
LDL cholesterol (mg/dL)	140.4 ± 37.9	135.2 ± 37.5
Leptin (ng/mL)	14.3 ± 14.0	13.3 ± 14.2
GPT/ALT (U/I)	26.1 ± 11.9	21.4 ± 8.83*
TSH (µU/mL)	1.33 ± 0.95	1.47 ± 1.21
Inflammatory parameters		
Ferritin (ng/mL)	169.8 ± 143.8	111.1 ± 83.9*
High-sensitivity CRP (mg/dL)	0.34 ± 1.08	0.16 ± 0.19
IL-6 (pg/mL)	2.82 ± 2.52	2.41 ± 1.35

Continuous variables: mean ± SD, unequal variance *t*-test/Student *t*-test, *P* values < .05 were regarded as statistically significant, statistical significant difference between groups: *P < .001.

Abbreviations: AUCglucose, area under the curve of plasma glucose; CRP, C-reactive protein; GLP-1, glucagon like peptide-1; GPT/ALT, alanine aminotransferase; HDL, high-density lipoprotein; HOMA-IR, homeostasis model assessment index for insulin resistance; LDL, low-density lipoprotein.

In the binomial logistic regression model, 2h-PG and fasting GLP-1 increased and 1h-PG decreased the odds of having an mp CS (Table 4). The model was statistically significant (χ^2^ (13) = 125,1, *P* < .001), resulting in a high proportion of explained variance (Nagelkerke's *R*^2^ = 0453 [22]). Overall, percentage of accuracy in classification was 84.5%, with a specificity of 93.8% and a sensitivity of 52.5%. The sensitivity analyses revealed similar results except that in the group without metS, GLP1 was not significant any more despite a similar odds ratio as in the analysis of the whole group (odds ratio, 1.056; *P* = .127, data not shown).

**Table 4.** Binary logistic regression model to determine the effect monophasic CS

	B	SE	*P*	Odds ratio	95% CI for Exp(B)	
					Lower bound	Upper bound
Sex (men)	0.716	0.462	.121	2.047	0.198	1.208
BMI (kg/m^2^)	0.106	0.077	.171	1.112	0.955	1.293
Waist circumference (cm)	−0.009	0.025	.728	0.991	0.945	1.040
1-h plasma glucose (mg/dL)	0.053	0.008	<.001	1.054	1.037	1.072
2-h plasma glucose (mg/dL)	−0.025	0.009	<.01	0.975	0.959	0.992
HOMA-IR (mg/dL)	−0.055	0.197	.781	0.947	0.643	1.393
GLP-1 (pM)	0.064	0.031	<.05	1.066	1.003	1.133
Triglyceride (mg/dL)	0.004	0.004	.376	1.004	0.996	1.012
HDL cholesterol (mg/dL)	0.020	0.012	.097	1.021	0.996	1.045
GPT/ALT (U/I)	−0.005	0.020	.789	0.995	0.957	1.034
Ferritin (ng/mL)	0.000	0.002	.963	1.000	0.997	1.004

Binomial logistic regression model adjusted for age and study center: χ^2^ (13) = 125.1, *P* < .001; Hosmer-Lemeshow-Test: χ^2^ (8) = 11.847, *P* > .05; Nagelkerke's *R*^2^ = 0.453. Overall percentage of accuracy in classification: 84.5%; specificity: 93.8%; and sensitivity: 52.5%.

Abbreviations: BMI, body mass index; CS, curve shape; GLP-1, glucagon like peptide-1; GPT/ALT, alanine aminotransferase; HDL, high-density lipoprotein.

## Discussion

In the present study, we analyzed the shape of the OGTT-curve in relation with anthropometric, metabolic, and inflammatory parameters in a well-defined group of healthy middle-aged and older people. The prevalence of mp (77.4%) was comparable to other investigations of OGTT CS that reported a rate of 57% to 87% mp [5, 7, 20]. Only 1 participant with an mp CS revealed a continuous rise curve. In the univariate analysis, men had a significantly higher prevalence of an mp CS than women. However, this sex difference disappeared in the binary logistic regression. This might be explained by the higher basal GLP-1 level and the increased 1-h plasma glucose of the male participants that were more closely associated to the CS than sex. A relationship between increasing age and less complex CS and thus a worse glucose metabolism profile has been previously described in healthy adult populations [5, 7, 16]. In contrast to these former studies, our data showed a similar rate of mp CS in both age groups. This might be explained by the aim of our study to enroll ∼50% of middle-aged persons with increased waist circumference what could have led to a higher proportion of mp in this age group. Consistent with this, the prevalence of metS in our cohort was nearly twice as high as in the general adult population in Germany (38.4 vs 19, 8%) [17].

In our sample, most anthropometric and metabolic variables were less favorable in participants with mp CS. This association between mp CS and prognostically less favorable metabolic parameters has already been described in several studies [5, 7, 16, 21]. An association of mp CS with impaired FPG and increased TG levels was described in studies in younger individuals (median age, 44 years) [15]. In the current study, AUCglucose was also higher in the mp group. The resulting higher glucose level has already been linked to a worse health status with more visceral fat, lower insulin clearance, and lower muscle insulin sensitivity in healthy, nonobese men with mp CS [4]. This might be in line with the higher GPT/ALT levels in our mp group. Some studies have reported that elevated GPT/ALT levels, even within the normal range, predict the development of type 2 diabetes mellitus (T2DM) and metS [23-25]. This association is probably caused by a slightly worsening hepatic ability to metabolize glucose [25].

It is well known that GLP-1 does improve glucose metabolism not only by slowing the gastric emptying process but also having a positive effect on blood glucose levels by its insulinotropic effect on ß cells and inhibition of glucagon secretion [26]. Therefore, we expected a lower fasting GLP-1 to be associated with mp shape. Contrary to our expectation, the fasting levels of GLP-1 were higher in participants with an mp CS. In line with this, increased GLP-1 levels were found to be associated with a higher degree of insulin resistance, hyperglycemia, hypertension, and dyslipidemia in overweight adolescents [27]. It is assumed that increased basal GLP-1 levels enlarge the pancreatic β-cell reservoir to compensate the greater need of insulin due to insulin resistance and to counteract weight gain [28]. In contrast to our results, a longitudinal study in adults aged >65 years found no difference in GLP-1 baseline levels between mono- and polyphasic curve shapes, but higher peak levels and AUC for GLP-1 in polyphasic participants [16]. A deterioration in glucose metabolism seems to be related to a worsening GLP-1-response [29]. Therefore, for GLP-1 lower basal as well as higher levels after glucose uptake are associated with better glucose metabolism.

Among the inflammatory parameters, only ferritin differed between the 2 CS groups. According to current research, higher ferritin levels, even within the normal range, might indicate an higher T2DM risk [30]. However, since ferritin also represents iron status, it is highly susceptible to false-positive results. All other inflammatory parameters—CRP, IL-6—were comparable in both subgroups. This could be due to the fact that the study population was healthy and had a low and uniform inflammation state. Nevertheless, this result was somehow unexpected, since especially IL-6 and CRP are associated with T2DM and have been considered as early markers for later progression [31, 32].

This study has certain strengths and limitations. It is a secondary analysis of a descriptive, observational, cross-sectional study with the aim of phenotyping healthy persons of different age groups comprehensively [15]. Persons with serious, but very common diseases in old age were excluded. This could have caused a significant bias in the study population, especially toward no significant differences for inflammatory parameters between the 2 subgroups. On the other hand, we have carefully examined a population group that could benefit from an early detection of a metabolic risk status. Although the OGTT was performed only once, it was previously shown that OGTT has a fairly consistent level of reproducibility [15]. Similarly, all biomarkers have been measured once at baseline, so there was no analysis possible about the further dynamic course. Furthermore, ß-cell function and insulin sensitivity could only be estimated by HOMA-IR and was not directly measured (eg, with the hyperinsulinemic euglycemic clamp method). In addition, GLP-1 levels were only measured in the fasting state, but not postprandially; this would have brought additional insights. GLP-1 was measured in EDTA-plasma without further inhibitors for stabilization what might have influenced the measured concentrations.

## Conclusion

In conclusion, we found no differences in OGTT CS between middle-aged and older persons, but a significant higher prevalence of mp CS in men compared to women. The results of our analysis clearly suggest an association between mp CS and prognostically less favorable anthropometric and metabolic parameters.

## Data Availability

The original contributions presented in the study are included in the article, further inquiries can be directed to the corresponding author.

## Abbreviations

2h-PG: 2-hour plasma glucose
ALT: alanine-amino-transferase
BMI: body mass index
BP: blood pressure
BW: body weight
CRP: C-reactive protein
CS: curve shape
FPG: fasting plasma glucose
GPT: glutamate-pyruvate-transaminase
HDL: high-density lipoprotein
HOMA-IR: homeostasis model assessment for insulin resistance
metS: metabolic syndrome
mp: monophasic
OGTT: oral glucose tolerance test
pp: polyphasix
T2DM: type 2 diabetes mellitus
TG: triglyceride
WC: waist circumference
